# Low-Level Laser Therapy for Temporomandibular Disorders: A Systematic Review with Meta-Analysis

**DOI:** 10.1155/2018/4230583

**Published:** 2018-05-10

**Authors:** Gang-Zhu Xu, Jie Jia, Lin Jin, Jia-Heng Li, Zhan-Yue Wang, Dong-Yuan Cao

**Affiliations:** ^1^Key Laboratory of Shaanxi Province for Craniofacial Precision Medicine Research, Research Center of Stomatology, College of Stomatology, Xi'an Jiaotong University, Xi'an, Shaanxi, China; ^2^Department of Neurosurgery, First Affiliated Hospital of Xi'an Medical University, Xi'an, Shaanxi, China; ^3^Department of Psychiatry, Xi'an Mental Health Center, Xi'an, Shaanxi, China

## Abstract

**Objectives:**

We systematically reviewed randomized controlled trials (RCTs) of the effect of low-level laser therapy (LLLT) versus placebo in patients with temporomandibular disorder (TMD).

**Methods:**

A systematic search of multiple online sources electronic databases was undertaken. The methodological quality of each included study was assessed using the modified Jadad scale, and the quality of evidence was evaluated using the Grading of Recommendations, Assessment, Development and Evaluation (GRADE) system.

**Results:**

A total of 31 RCTs were included. Total modified Jadad scale scores showed that the methodological quality was high in 30 studies and low in 1 study. Combining data from all clinically heterogeneous studies revealed positive effects of LLLT on pain relief, regardless of the visual analogue scale (VAS) score or the change of VAS score between the baseline and the final follow-up time point, while dosage analyses showed discrepant results about the effects of high or low doses for patients with TMD. Follow-up analyses showed that LLLT significantly reduced pain at the short-term follow-up. Temporomandibular joint function outcomes indicated that the overall effect favored LLLT over placebo.

**Conclusion:**

This systematic review suggests that LLLT effectively relieves pain and improves functional outcomes in patients with TMD.

## 1. Introduction

Temporomandibular disorder (TMD) is a set of clinical conditions that includes disorders of the temporomandibular joint (TMJ) and/or the masticatory muscles [1]. The most common symptoms are pain, joint noises, and restricted mandibular movement [2]. A variety of other symptoms may occur, such as tinnitus, abnormal swallowing, and hyoid bone tenderness [3, 4]. These symptoms compromise quality of life (QoL) [5], sleep [6], and the psychological well-being, leading to anxiety, stress, depression, and a negative effect on social function, emotional health, and energy level [7]. The incidence of signs and symptoms of TMD varies from 21.5% to 50.5%, and they occur more frequently among women than men [8–10].

The etiopathogenesis of TMD remains unclear. In general, it is thought that the origin of TMD is multifactorial, including biomechanical, neuromuscular, biopsychosocial, and biological factors [11]. Therefore, the mainstay of treatment for TMD is a multidisciplinary approach that includes physical therapy modalities such as manual therapy [12], electrotherapy [13], ultrasound [14], transcutaneous electrical nerve stimulation (TENS) [15], or laser therapy [16].

Among the various physical therapy modalities, low-level laser therapy (LLLT) has recently been put under the spotlight because of its easy application, short treatment time, and few contraindications. Many prospective clinical trials have been performed to evaluate the efficacy of LLLT. However, the results have been controversial [16–21]. Some authors have reported the superiority of LLLT over placebo [16, 20, 21], while others have found no significant differences between LLLT and placebo [17–19].

Over the past recent years, a number of systematic reviews with or without meta-analysis have analyzed the efficacy of LLLT for TMD [2, 22–27]. Based on the included studies, which were all published before 2010, four systematic reviews concluded that there was no definite evidence to support the use of LLLT in the management of TMD [22–25]. On the contrary, one meta-analysis published in 2014 concluded that applying LLLT to the masticatory muscle or joint capsule has a moderate analgesic effect on TMJ pain [2]. In 2015, another meta-analysis provided evidence that using LLLT has limited efficacy in reducing pain, but can significantly improve the functional outcomes of patients with TMD [26]. However, there is no solid evidence to support or refute LLLT for TMD.

Since the latest published meta-analysis, many new randomized controlled trials (RCTs) have been conducted, which may accumulate evidence on the use of LLLT for TMD [4, 28–31]. Therefore, in this systematic review with meta-analysis, we reevaluated the effect of LLLT versus placebo in patients with TMD. The results of this study may provide practical recommendations for clinical physicians who treat patients with TMD.

## 2. Methods

### 2.1. Search Strategy and Selection Criteria

This review was conducted in accordance with the Preferred Reporting Items for Systematic Reviews and Meta-Analyses (PRISMA) statement guidelines [32, 33] and Cochrane handbook for systemic reviews [34]. We systematically searched PubMed, EMBASE, CINAHL, ClinicalTrials.gov, the Cochrane Library database, AMED, Toxline, PEDro, ProQuest Digital Dissertations, PsycBite, SCOPUS, Current Contents Connect, Web of Science, and the WHO Trial Registry for RCTs comparing LLLT with a placebo intervention in patients with TMD.

The following search terms were used: “temporomandibular disorder(s)” OR “temporomandibular joint disorder(s)” OR “temporomandibular joint dysfunction” OR “TMJ disorder(s)” OR “TM disorder(s)” OR “temporomandibular joint pain” OR “temporomandibular pain” OR “TM pain” OR “TMJ pain” OR “TMD” OR “temporomandibular osteoarthritis” OR “myofascial pain” OR “craniomandibular disorder(s)” OR “mandibular dysfunction” AND “laser” OR “laser therapy” OR “low level laser therapy” OR “low intensity laser therapy (LILT)” OR “low energy laser therapy (LELT)” OR “LLLT” OR “infrared (IR) laser” OR “IR laser” OR “diode laser” OR “helium-neon laser” OR “HeNe laser” OR “gallium-arsenide laser” OR “GaAs laser” OR “gallium-aluminium-arsenide laser” OR “GaAlAs laser.” The last search was performed on May 16, 2017.

Inclusion criteria were as follows: (1) RCTs involving patients with TMD; (2) articles published or informally published in English or Chinese; and (3) primary studies or studies in which LLLT was compared with placebo or sham laser, with similar appearance to the active treatment but without laser irradiation, in patients with TMD; and (4) studies of LLLT for myogenous or arthrogenous temporomandibular pain, or both, regardless of age and gender. Studies including cointerventions were allowed if applied equally to both the LLLT and placebo groups.

Exclusion criteria were as follows: (1) nonrandomized or crossover studies; (2) total number of study participants (in the LLLT and placebo groups combined) less than 10; (3) meeting abstracts that did not report data for the outcomes of interest; and (4) studies involving patients with systemic diseases (i.e., fibromyalgia and rheumatoid arthritis) or pain not related to TMD (i.e., neuralgia, toothache, and psychological disturbances).

### 2.2. Study Selection

Two independent reviewers (Xu and Jia) initially screened and identified relevant titles and abstracts. Full-text articles were obtained for all eligible studies, and these were assessed independently by Jin and Li against an inclusion and exclusion checklist. Disagreements were resolved by discussion until consensus was reached; if this approach failed, a third party (Cao) was consulted. The reference lists of all retrieved studies were manually examined to identify any studies missed by the electronic literature search. We also contacted all principal investigators or corresponding authors of the identified studies for additional information where necessary.

### 2.3. Outcome Measures

The primary outcome of interest was pain intensity, as expressed by visual analogue scale (VAS) score (at the final follow-up time point) or the change of VAS score (between the baseline and the end of the follow-up) in the LLLT and placebo groups.

The secondary outcomes included the change of TMJ function between the baseline and the end of the follow-up, oral function (masticatory performance), electromyographic (EMG) activity, adverse effects, pressure pain threshold (PPT), joint noises, tinnitus, quality of life (QoL), and psychological satisfaction in the LLLT and placebo groups.

TMJ function was assessed in terms of maximum active vertical opening (MAVO), maximum passive vertical opening (MPVO), lateral excursion (LE), and protrusion excursion (PE), expressed in millimeters.

### 2.4. Data Extraction

We used data from the longest follow-up time point for each trial. Data were extracted and crosschecked independently by Li and Wang using a standard data extraction form that contains general information (authors, publication year), subject number, treatment-related information, and relevant clinical outcome data. Authors were contacted to clarify further information where necessary. In three RCTs that examined more than one laser dose, the placebo group was divided into two equal-sized groups to avoid “double counting” to allow inclusion of two independent comparisons within the meta-analysis [30, 35, 36].

Data from the included studies were pooled for further meta-analysis where appropriate. If available, means and standard deviations for outcome measures were extracted or calculated based on the published data with RevMan 5.0 software as supplied by the Cochrane Collaboration. Means and standard deviations were used to calculate mean differences (MDs) and 95% confidence intervals (CIs) in the meta-analysis.

### 2.5. Assessment of Methodological Quality

All included studies were assessed for methodological quality using the modified Jadad scale [37]. Two reviewers (Xu and Jia) performed the assessment independently, and discrepancies between reviewers were resolved by consensus. Studies achieving four or more points (from a maximum of eight) were considered to be of high quality, while studies scoring below four were considered to be of low quality.

### 2.6. Statistical Analysis

Dichotomous outcomes were expressed as relative risks (RRs) and continuous outcomes were expressed as the weighted mean differences (WMDs), both were presented with 95% CIs. Pooled effect sizes were based on the results of pain intensity (assessed by VAS) as well as MAVO, MPVO, LE, and PE values in millimeters. Revman 5.0 Software was used to summarize the effects and to construct the forest plots for all comparisons. Heterogeneity was examined according to the *I* ^2^ statistic alongside the chi-squared test; if *I* ^2^ was greater than 50%, the random-effects model was applied [38]. Qualitative analysis was performed if studies failed to provide data to be pooled for analysis. Publication bias was assessed by examination of funnel plots for primary outcomes. A symmetric funnel plot represented lower risk of bias and vice versa. Because interstudy heterogeneity precluded a meta-analysis in some outcomes, narrative synthesis of related studies was employed.

### 2.7. Subgroup and Sensitivity Analysis

Subgroup and sensitivity analysis were planned in the presence of heterogeneity. Subgroup analysis was performed to evaluate the effect of the intervention at different laser dosages and follow-up periods (short-term and long-term effects). Sensitivity analysis was performed for testing the robustness of the pooled effect size where appropriate. Effects were examined according to methodological quality, to ensure that the analysis was not biased by a low-quality study or a study with a large population.

### 2.8. Evaluation of Quality of Evidence

The quality of evidence was evaluated using the Grading of Recommendations, Assessment, Development and Evaluation (GRADE) system [39], which is based on five domains (limitations of the study design, inconsistency, indirectness, imprecision, and publication bias).

## 3. Results

### 3.1. Search Results

**Figure 1:**
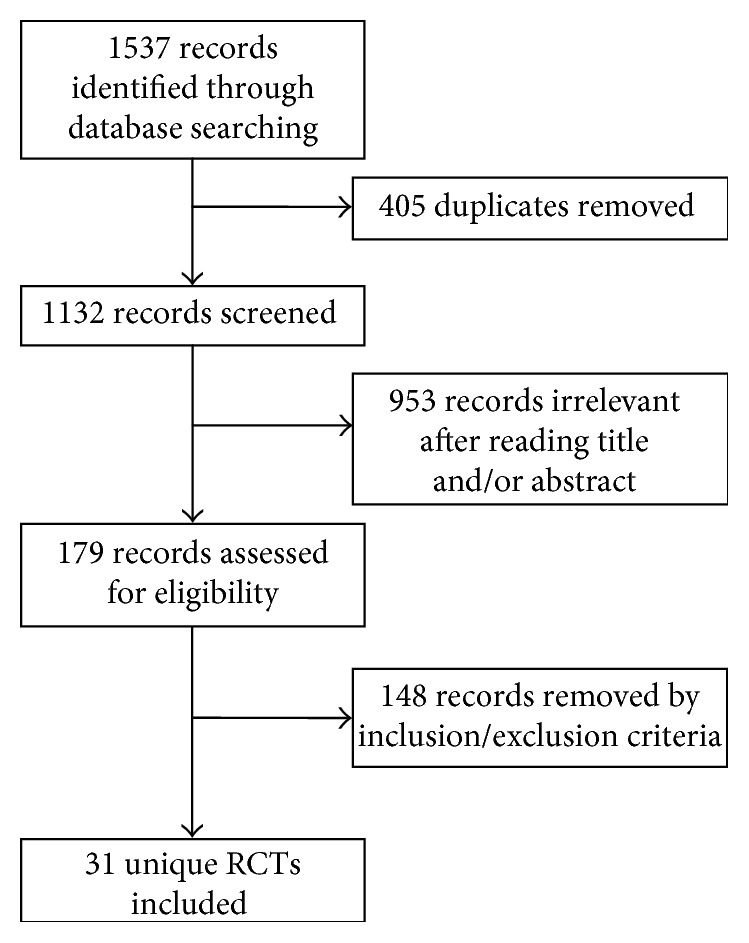
The study selection process for the systematic review.

The study selection process is shown in Figure 1. A total of 1537 records were identified from searches. No unpublished manuscripts were identified. After excluding 1506 records, a total of 31 articles that met the inclusion criteria were included in the present systematic review.

### 3.2. Characteristics of the Included Studies

General information and technical features of the included studies are summarized in Tables 1 and 2, respectively. All studies were RCTs published in English, except one Chinese study. Participants received a total of 3 to 20 treatment sessions. There were seven different types of laser among the 31 included studies. Gallium-aluminium-arsenide laser (GaAlAs) was applied in 20 studies [1, 16, 17, 19–21, 28, 29, 31, 35, 36, 40–48], gallium-arsenide laser (GaAs) in six studies [30, 49–53], and neodymium-doped yttrium aluminum garnet (Nd:YAG) in two studies [4, 54]. Helium-neon laser (HeNe) [18], indium-gallium-aluminum-phosphide laser (InGaAlP) [53], and diode laser [4] were applied each in one study. The laser type was not mentioned in two studies [55, 56]. The shortest wavelength of laser was 632.8 nm and the longest was 1064 nm. Laser dosage varied from 1.5 J/cm^2^ to 112.5 J/cm^2^; four studies did not report the dosage [20, 21, 31, 50].

Two studies including cointerventions applied equally to both LLLT and placebo groups: in one study, LLLT was combined with piroxicam [28]; in the other study, it was combined with oral motor (OM) exercises [40]. Two studies investigated the combination of two types of laser: one study applied InGaAlP (660 nm) and GaAs (890 nm) [53], while the other applied Nd:YAG (1064 nm) and diode laser (810 nm) [4]. One study combined GaAlAs at two wavelengths (650 nm/830 nm) [21]. There were four studies using only one laser type, but at two or three laser dosages [35, 36, 44, 45]. There was one study which applied one type of laser, but at two application sites [30]. The majority of the included studies compared LLLT and placebo groups, except for four studies involving other interventions, namely, ibuprofen [20], occlusal splint [54], needling [55], and physiotherapeutic and drug protocol (PDP) [47]. The final follow-up time point varied from immediately to 3 months after completing the treatment. Application sites were generally the TMJ and/or temporomandibular muscles. One study added remote acupuncture points [16].

The majority of the included studies provided pain intensity data. Thirteen RCTs investigated mouth opening (MO) [17, 19–21, 28, 30, 36, 44, 46, 49, 51, 55, 56], nine focused on LE [17, 19–21, 30, 36, 46, 49, 51], six focused on PE [17, 19, 21, 30, 36, 51], seven focused on PPT [19, 29, 30, 35, 48, 51, 55], three focused on joint noises [49, 50, 56], two focused on masticatory efficiency [1, 29], and one focused on subjective tinnitus [4]. Three studies applied EMG as a study parameter [35, 45, 55].

### 3.3. Quality Assessment

A summary of the quality assessment using the modified Jadad scale scores is shown in Table 1. Total scores showed that the quality of 30 studies was high, with a minimum of 4 points and a maximum of 8 points. One study had low quality (3 points) [50] (Supplementary Material Appendix A).

### 3.4. Effects of Laser Therapy

The 31 RCTs showed mixed results, as reported by the authors, with two-thirds reporting positive effects favoring LLLT and one-third reporting inconclusive results or no effect. Twenty-two studies provided sufficient data to calculate effect sizes for key outcome measures and were included in the meta-analysis. Subgroup analysis was performed for laser dose and follow-up period using the random-effects model. Studies were subcategorized into low dosage (≤50 J/cm^2^) versus high dosage (>50 J/cm^2^) and into short-term follow-up (≤2 weeks) versus long-term follow-up (>2 weeks). For all studies, we only collected data from the final follow-up time point.

### 3.5. Primary Outcomes

All 22 studies, except three [4, 40, 47], used VAS to assess pain as one of the primary outcome measures. However, as a result of data detectability, only 19 studies were subjected to meta-analysis.

#### 3.5.1. VAS Score

Seventeen of the included studies provided VAS scores at the final follow-up time point. Meta-analysis of data from 643 participants across 17 studies indicated a statistically significant reduction in total pain scores in LLLT versus placebo groups. The overall effect for pain favored LLLT (WMD = −14.05; 95% CI = −25.67 to [−2.43]; *P*=0.02; *I* ^2^ = 96%), yet with substantial heterogeneity. Subgroup analysis showed significant differences between LLLT and placebo groups at high dosage (WMD = −10.42; 95% CI = −19.67 to [−1.17]; *P*=0.03; *I* ^2^ = 51%) and unknown dosage (WMD = −33.75; 95% CI = −57.18 to [−10.33]; *P*=0.005; *I* ^2^ = 98%). However, there were no significant differences between the two groups at low dosage (WMD = −9.22; 95% CI = −18.78 to 0.34; *P*=0.06; *I* ^2^ = 85%) (Supplementary Material Appendix B).

There were significant differences between the two groups at the short-term follow-up (WMD = −14.66; 95% CI = −21.04 to [−8.29]; *P* < 0.00001; *I* ^2^ = 71%). However, LLLT failed to show significant favorable effects on pain scores at long-term follow up compared to the placebo (WMD = −14.84; 95% CI = −35.35–5.68; *P*=0.16; *I* ^2^ = 97%) (Supplementary Material Appendix B).

#### 3.5.2. Mean Difference of VAS Score

The mean difference of VAS score (change scores from baseline) between the baseline and the final follow-up time point was used [17, 42, 49, 53]. When the data were missing, we calculated them using the published relevant data with RevMan 5.0 software [1, 16, 18–21, 28, 30, 31, 35, 41, 43, 50, 51, 54]. Analysis of data from 679 subjects (19 studies) revealed a significant difference between the LLLT and placebo groups (WMD = 15.43; 95% CI = 3.61–27.26; *P*=0.01; *I* ^2^ = 98%). Subgroup analysis showed significant differences at low dosage (weighted mean difference = 15.09; 95% CI = 5.37–24.80; *P*=0.002; *I* ^2^ = 93%) and unknown dosage (WMD = 36.31; 95% CI = 10.63–61.98; *P*=0.006; *I* ^2^ = 99%). However, there were no significant differences between the two groups at high dosage (WMD = 5.52; 95% CI = −5.52 to 16.56; *P*=0.33; *I* ^2^ = 80%) (Supplementary Material Appendix B).

In term follow up subgroup, similar to VAS score, there was significant differences between the two groups in the short-term follow up subgroup (WMD = 17.66; 95% CI = 9.94–25.38; *P* < 0.00001; *I* ^2^ = 90%), but not at long-term follow up compared to the placebo (WMD = 13.85; 95% CI = −7.73 to 35.43; *P*=0.21; *I* ^2^ = 99%) (Supplementary Material Appendix B).

#### 3.5.3. Sensitivity Analysis

The pooled results were in favor of LLLT based on the above-mentioned main findings (see Supplementary Material Appendix C), after excluding low-quality [50] and/or extreme value and maximal weight value (large population) studies [20].

### 3.6. Secondary Outcomes

#### 3.6.1. Functional Outcomes

The secondary outcomes included the change of TMJ function from baseline to the end of the follow-up in LLLT and placebo groups. TMJ function was assessed in terms of MAVO, MPVO, LE, and PE. These four outcomes all indicated that the overall effect favored LLLT over placebo: MAVO (WMD = 6.37; 95% CI = 2.82–9.93; *P*=0.0004; *I* ^2^ = 95%), MPVO (WMD = 6.96; 95% CI = 1.99–11.93; *P*=0.006; *I* ^2^ = 92%), LE (WMD = 3.52; 95% CI = 2.63–4.40; *P* < 0.00001; *I* ^2^ = 90%), and PE (WMD = 1.77; 95% CI = 0.09–3.45; *P*=0.04; *I* ^2^ = 95%).

Most of these outcomes showed significant differences between the LLLT and placebo groups, except for MAVO and PE at low dosage and PE at unknown dosage. In addition, there were no MPVO data in the high-dosage subgroup. All data of TMJ function in follow-up subgroups showed significant differences between the LLLT and placebo groups, except for MAVO at the short-term follow-up (Supplementary Material Appendix D).

#### 3.6.2. PPT

Seven studies investigated pain by measuring PPT [19, 29, 30, 35, 48, 51, 55], expressed as mm, kpa, or kg/cm^2^. It is impossible to estimate the overall effect size across the different scales. Four studies showed a significant change [29, 30, 35, 51], while two reported no change of PPT in the LLLT group compared to the placebo group [19, 48]. In another study [55], a significant improvement was observed in the LLLT group only at a dosage of 4 J/cm^2^ (*P*=0.0156), but not at 8 J/cm^2^ (*P*=0.4688).

#### 3.6.3. EMG Activity

Three studies measured EMG activity before and after treatment [35, 45, 55]. LLLT did not promote any changes in EMG activity [45, 55]. However, in another study [35], EMG records in the maximum voluntary clenching (cEMG) of the MLILT (a modified high-energy LILT) group were significantly higher after the final treatment than those of the placebo group (*P*=0.022, 95% CI = 5.96–68.66 microV), but there was no significant difference of cEMG between the CLILT (a conventional low-energy LILT) and placebo groups.

#### 3.6.4. Oral Function Outcome Measures

Masticatory efficiency was evaluated in two studies [1, 29]. However, the used methods were different, for the results were considered inadequate for meta-analysis. Although both studies indicated that masticatory performance might be better after LLLT treatment, only one study [29] showed a significant improvement in masticatory performance in the LLLT group at the end of treatment compared with baseline values (*P* < 0.01).

#### 3.6.5. Joint Noises

Three studies investigated joint noises following LLLT [49, 50, 56]. Two studies reported that LLLT could not reduce joint noises [49, 56]. Lassemi et al. reported LLLT could reduce “Click” compared to placebo [50], but the conclusion is questionable due to the low quality of the methodology.

#### 3.6.6. Tinnitus

One study evaluated subjective tinnitus [4]. The study applied two types of LLLT in bilateral subjective tinnitus with TMD, namely, LLLT with Nd:YAG (1064 nm) and LLLT with a diode laser (810 nm). Both Nd:YAG and diode laser were effective for the treatment of subjective tinnitus related to TMD.

#### 3.6.7. QoL and Psychological Satisfaction

None of the included studies reported QoL or psychological satisfaction as an outcome measure.

### 3.7. Adverse Effects

Nine of the 31 included studies reported no adverse effects related to laser application during or after the treatment period [4, 16, 28, 30, 47, 51–54]. The other studies lacked information regarding the adverse effects of laser exposure.

### 3.8. Publication Bias

Considering the heterogeneity of the studies, funnel plots were drawn according to different outcome measures. Visual assessment of funnel plots did not show considerable asymmetry in pain (VAS score), LE, and PE, indicating that the publication-related bias was low for these outcomes. However, there was asymmetry in MAVO and MPVO outcomes, indicating that the publication bias was high for these two outcomes (Supplementary Material Appendix E).

### 3.9. Quality of Evidence

The present meta-analysis investigated a total of six types of outcomes (including 29 subgroup analyses stratified by laser dosage and follow-up period) about pain intensity and mandibular function. The GRADE assessment of the level of evidence for these outcomes is shown in Supplementary Material Appendix F. The quality of the evidence was judged to range from very low to moderate. All domains affected low grades except indirectness.

## 4. Discussion

This systematic review and meta-analysis summarized RCTs that compared the effect of LLLT with placebo for the treatment of TMD. The results of the studies indicated that LLLT was effective in reducing TMD pain compared to placebo. In addition, LLLT could improve functional outcomes. Combining data from all clinically heterogeneous studies demonstrated positive effects of laser on pain relief, regardless of VAS score or the change of VAS score between the baseline and the final follow-up time point, while dosage subgroup analyses showed discrepant results about high or low dosage for TMD patients. The follow-up subgroup analysis showed more consistent results, suggesting LLLT significantly reduced pain at the short-term follow-up both in the VAS score and the change of VAS score. However, there was no significant difference at the long-term follow-up between LLLT and placebo. TMJ function outcomes, assessed in terms of MAVO, MPVO, LE, and PE (the changes between baseline and the end of follow-up) indicated that the overall effect favored LLLT over placebo. Most (five out of 7) studies indicated PPT improved by LLLT, but most (two out of 3) studies showed no change in EMG activity.

The use of LLLT has been seen as a complementary option for the treatment of TMD [16, 20, 21, 26] due to its analgesic, anti-inflammatory, and regenerative effects with no reported adverse effects and good acceptance by patients [16, 28, 30, 51, 53, 54, 57, 58]. In view of the lack of robust evidence about the effects of LLLT on TMD, recent systematic reviews did not reach a consensus [2, 22–27]. Here, we update the clinical evidence for the effects of LLLT on TMD.

Our results regarding the analgesic effect of LLLT are consistent with findings of Chang et al. [2], in contrast to those of Chen et al. [26], but the results regarding functional outcomes (motion) were in accordance with those of Chen et al. [26]. The strengths of our systematic review are the larger number of studies and the inclusion of the most recent publications since the last review in the subject.

As pain is the principal complaint of patients with TMD, pain is the most common reason why patients with TMD seek medical help. Pain occurs at any stage of TMD and pain reduction contributes to ameliorating jaw motion [26, 59], chewing [60], and masticatory performance [29]. Therefore, our primary outcome measure was pain intensity. Given the heterogeneity of the included studies, meta-analyses were performed using subgroups of studies according to the dosage and follow-up time. Although the overall effects of LLLT on pain were positive in both the VAS score and the change of VAS score, subgroup analysis reached contrary conclusions for high dosage versus low dosage in these two parameters. It is difficult to draw precise conclusions regarding an effective dosage window from these studies due to the contrary conclusions from subgroup analysis (analyzed by actual VAS score or the change of VAS score) and the wide dosage range employed. Four of the included studies [35, 36, 43, 45] compared high dosage with low dosage, but only one study [35] showed the superiority of high dosage, while the others showed no differences between the two dosages. The mechanism underlying the therapeutic effects of LLLT is under debate [61, 62]. The magnitude of the laser effect seems to also depend on the dosage of laser [63]. Bjordal et al. [64] believed that the controversy on the efficacy of LLLT on TMD laid in the disagreement on the dosage of laser. Laser acupuncture has been suggested to be a dosage-dependent modality [65, 66], suggesting that the energy delivered to the target point by laser acupuncture has to reach a threshold in order to produce a desired effect. Thus, the dosages used in the included studies may explain the observed differences in outcomes.

With regard to the relationship between laser effectiveness and follow-up period, Law et al. found that long-term follow-up effects increased in three types of musculoskeletal disorders (myofascial pain/musculoskeletal trigger points, lateral epicondylitis, and temporomandibular joint pain) [67]. Pooled effect sizes were doubled during the follow-up period compared to those at the end of intervention, suggesting that laser may have delayed or long-lasting effects. However, our follow-up subgroup analysis showed a more consistent result, contrary to Law et al., both of pain evaluation methods showing significant differences only in short-term follow up between the laser and placebo groups. In one trial conducted by Carli et al. [28], laser, piroxicam, and placebo significantly improved the VAS score. An evaluation at 30 days after the end of the treatment showed that the laser did not have a residual effect, and piroxicam was more effective than the laser to reduce the level of muscular pain in patients with TMJ arthralgia. These results illustrate that laser may have a short-lived effect, which is consistent with our follow-up subgroup analysis data. However, because of the high degree of heterogeneity, this finding needs to be explored in further research. Nonetheless, the present systematic review and others support the continued use of laser for treating TMD/musculoskeletal pain.

Besides the subjective pain assessments, the objective clinical outcomes include TMJ function, PPT, EMG, and masticatory performance. The overall effects in MAVO, MPVO, LE, and PE favored LLLT over placebo. In most studies, LLLT increased PPT, but did not affect EMG activity. Only one study [29] showed that LLLT improved masticatory performance at the end of treatment compared with baseline values, but other study [1] showed no significant effects.

Skin surface application of laser (trigger points/tender points) was used in most of the included studies, external auditory meatus was used in some studies [4, 41, 52], and only one study added remote application sites at acupuncture points [16]. We are unable to perform analysis on different sites for the variety and the complexity of these application sites.

It is worth to mention that using the combination of two wavelengths [21, 53] yields positive result. Shirani et al. [53] combined GaAs and InGaAlP lasers, usually applied for deep-lying disorders and superficial disorders, respectively. Another study [21] applying GaAlAs at 650 nm and 830 nm also obtained good effects, implying that the combination of two laser wavelengths may be beneficial to patients with TMD. During TMD treatment with LLLT, the variability of laser type, frequency, dosage, exposure time, application area, number of laser sessions, and therapy duration may increase heterogeneity in effects. Thus, the findings of clinical studies must be interpreted against the background. Using proper laser parameters is important to obtain better effects, as suggested by Law et al. [67] Additionally, due to the multifactorial etiology of TMD, including biopsychosocial and biological factors [11], about one-third of the patients report eating problems and feelings of depression or dissatisfaction with life [68]. However, none of the included studies focused on psychological assessment. TMD diagnosis has been standardized based on research diagnostic criteria for temporomandibular disorders (RDC/TMD) that constitute a multidimensional diagnostic research tool adopted worldwide [69, 70]. This standardization has improved reproducibility among clinicians and has facilitated the comparison of results among researchers [16, 28, 44]. It is important to establish standardized therapy regimens about TMD through evaluating relevant behavioral, psychological, and psychosocial factors (e.g., pain status variables, depression, nonspecific physical symptoms, and disability levels) [69, 70]. In addition, the effect of laser can be further evaluated by adding QoL and patient satisfaction as outcome measures.

Nine of the 31 included studies explicitly stated that no adverse effects were observed [4, 16, 28, 30, 47, 51–54]. The other studies lacked information regarding the adverse effects of laser exposure. Nevertheless, it is thought that LLLT is safe. LLLT is noninvasive and has few or none adverse effects, which may contribute to increased patient comfort.

This systematic review has some limitations. First, the methodological quality varied among the included studies. Second, there was a high degree of heterogeneity because of differences in TMD diagnosis, laser style, laser parameters, treatment regimens, outcome measurements, and follow-up time, which hindered some comparisons between studies. Furthermore, discrepancies also existed in the inclusion and exclusion criteria. Third, although we tried to obtain full data, some data were missing because some of the studies reported continuous variables such as MAVO or MPVO without SD, while others used box plots or histograms to represent the data. Moreover, most of the included studies had small sample sizes (≤60 subjects), which limited the generalizability of the conclusions. Fourth, although a systematic search of multiple databases was undertaken, some unpublished grey literature might have been missed. In addition, some of the included studies were not used in the meta-analysis. Thus, potential publication bias and selection bias could not be eliminated. We only included English and Chinese language articles, which could induce a language bias. Pooled analysis of all kinds of TMD (muscular origin, articular origin, or a combination of both) degraded the level of conclusion because of the poor description of TMD type in some studies. Given the above reasons, the findings from this study should be interpreted cautiously.

## 5. Conclusion

The results of this systematic review and meta-analysis are encouraging. Despite the above-mentioned limitations, the overall effect illustrated that LLLT effectively relieves pain in the treatment of TMD. LLLT may induce a short-term effect only, but the existing evidence does not allow us to determine an effective dosage window. Moreover, LLLT also improves the functional outcomes in TMD. In view of the high discrepancy among the included studies, this systematic review highlights the need for more well-designed RCTs with larger sample sizes to evaluate the efficacy of LLLT. Future research should carefully define the study population and provide the rationale for the parameters chosen. This would facilitate not only replication in the clinical setting, but also improve trial homogeneity and allow data to be pooled for meta-analysis. Furthermore, it is necessary to examine different laser parameters, treatment regimens, evaluation times, and outcome measures because it is noninvasive, safe, easy-to-use, and cheap.

## Figures and Tables

**Table 1:** General information and modified Jadad score in the included trials.

Study	Research location	*n*	Treatment design	Aspect evaluated	Evaluations/follow-up	Evaluation methods	Modified Jadad score
Conti [17]	Brazil	20	Laser (10) versus placebo (10)	PI, mandibular function	After each treatment	VAS, MO, LE, PE	5
Kulekcioglu [49]	Turkey	35	Laser (20) versus placebo (15)	PI, mandibular function, joint sounds,	Before, after, and 1 month after treatment	VAS, MO, LE, auscultation	5
Venancio [19]	Brazil	30	Laser (15) versus placebo (15)	PI, mandibular function, pain sensitivity	Immediately before the first, third, and fifth treatment sessions, and at the follow-up appointments after 15, 30, and 60 days of the end of treatment	VAS, MO, LE, PE, PPT	5
Mazzetto [41]	Brazil	48	Laser (24) versus placebo (24)	PI	Before treatment, after the 4th and 8th applications, and 30 d after the last application.	VAS	6
Cunha [42]	Brazil	40	Laser (20) versus placebo (20)	PI, TMD status	Before treatment and after the last treatment	VAS, DI, CMI, palpation index	5
Carrasco [1]	Brazil	14	Laser (7) versus placebo (7)	PI, masticatory efficiency	Before treatment, after the 8th application, 30 days after the last application	VAS, colorimetric capsule method	5
Emshoff [18]	Austria	52	Laser (26) versus placebo (26)	PI	Before treatment and 2, 4, and 8 weeks after the first laser therapy	VAS	7
Lassemi [50]	Iran	48	Laser (26) versus placebo (22)	PI, joint sounds	Before treatment, immediately, 2 and 4 days after treatment	VAS, stethoscope	3
Carrasco [43]	Brazil	60	Laser (30, 3 parameter groups, 10 in each group) versus placebo (30)	PI	Before treatment, after the 4th and 8th applications, 15 days and 1 month after the last application	VAS	5
Shirani [53]	Iran	16	Laser (the combination of two wavelengths, 8) versus placebo (8)	PI	Before and immediately after treatment, 1 week after treatment, and on the day of feeling complete pain relief	VAS	6
Marini [20]	Italy	99	Laser (39) versus ibuprofen versus (30) placebo (30)	PI, mandibular function, morphologic structural analysis of TMJ	PI at baseline, 2, 5, 10, and 15 days after treatment. Mandibular function at baseline, 15 days and 1 month after treatment. MRI at baseline and at the end of the treatment.	VAS, MO, LE, MRI	6
Sattayut [35]	England	30	Low energy laser (10) versus high energy laser (10) versus placebo (10)	PI, pain sensitivity, mandibular movements, EMG activity	Baseline and 1, 3, 5, and 8 days after treatment	VAS, PPT, EMG, McGill pain questionnaire	6
Silva [36]	Brazil	45	Low energy laser (15) versus high energy laser (15) versus placebo (15)	PI, madibular movements	Before treatment, immediately after the first, fifth, tenth treatments, and 5 weeks after completing the applications	VAS, MO, LE, PE	6
Ferreira [16]	Brazil	40	Laser (20) versus placebo (20)	PI	Before intervention, monthly until intervention completed	VAS	7
Ahrari [44]	Iran	20	Laser (10) versus placebo (10)	PI, madibular movements	Before intervention, after six applications, at the end of treatment, and 1 month after the last application	VAS, MO	6
Demirkol [54]	Turkey	30	Laser (10) versus occlusal splint (10) versus placebo (10)	PI	Before treatment, immediately and 3 weeks after treatment	VAS	4
Röhlig [51]	Turkey	40	Laser (20) versus placebo (20)	PI, functional examination, pain sensitivity	Before treatment and after the last applications	VAS, MO, LE, PE, PPT	8
Wang [21]	China	42	Laser (21) versus placebo (21)	PI, functional examination	Before treatment, immediately, 1 month and 2 months after treatment	VAS, MO, LE, PE	5
Carli [28]	Brazil	32	Laser + piroxicam (11) versus laser + placebo piroxicam (11) versus placebo laser + piroxicam (10)	PI, functional examination	Before treatment, after the first, second, third, and fourth treatment sessions, and 30 days after last treatment.	VAS, MO	8
Fornaini [31]	Italy	24	Laser (12) versus placebo (12)	PI	Before treatment, 1 and 2 weeks after treatment	VAS	5
Sancakli [30]	Turkey	30	Laser I (10) versus laser II (10) versus placebo (10)	PI, mandibular mobility, pain sensitivity	Before treatment and after the completion of therapy	VAS, MO, LE, PE, PPT	7
Frare [52]	Brazil	18	Laser (10) versus placebo (8)	PI	Before and immediately after all sessions of laser applications	VAS	4
Venezian [45]	Brazil	48	(1): Laser I (12) versus placebo I (12)	PI, EMG activity	PI: before treatment, immediately and 30 days after treatment	VAS, EMG	6
			(2): Laser II (12) versus placebo II (12)		EMG: before and immediately after treatment		
Mazzetto [46]	Brazil	40	Laser (20) versus placebo (20)	PI, mandibular movements	Before treatment, immediately, 7 and 30 days after applications	VAS, MO, LE	4
Uemoto [55]	Brazil	21	Laser (7) versus needling group (7) versus placebo (7)	PI, EMG activity, pain sensitivity, madibular movements	Before treatment, after four sessions with intervals ranging between 48 and 72 h	VAS, EMG, PPT, MO	4
Madani [56]	Iran	20	Laser (10) versus placebo (10)	PI, madibular movements, joint sounds	Before treatment, after 6 and 12 applications and 1 month after last application	VAS, MO, perceiving joint sounds by the fingertips	6
Maia [29]	Brazil	21	Laser (12) versus placebo (9)	PI, masticatory performance, pain sensitivity	MP and PPT, before treatment, at the end of treatment and 30 days after treatment	VAS, optical test material, PPT	5
					VAS, at the same time as above and was also measured weekly		
Cavalcanti [47]	Brazil	60	Laser (20) versus PDP (20) versus placebo (20)	Presence or absence of pain	Before treatment, at each week till the fourth week after treatment	Muscle tenderness palpation and the questionnaire of fonseca	4
Magri [48]	Brazil	91	Laser (31) versus placebo (30) versus control (30)	PI, pain sensitivity, the sensory and affective dimensions of pain	Before treatment, after each treatment and 30 days after last treatment	VAS, PPT, SF-MPQ	7
Demirkol [4]	Turkey	46	Nd:YAG laser (15) versus diode laser (16) versus placebo (15)	The severity of the tinnitus	Before treatment, immediately and 1 month after treatment	VAS	4
Machado [40]	Brazil	82	GI: laser + OM exercises (21) versus GII: pain relief strategies + OM exercises (22) versus GIII laser placebo + OM exercises (21) versus GIV: laser (18)	PI, TMD severity, and orofacial myofunctional status	Before treatment, immediately and 3 months after last treatment	ProTMDmulti-part II questionnaire, orofacial myofunctional evaluation with scores	5

CMI: craniomandibular index; DI: dysfunction index; EMG: electromyography; LE:_lateral excursion; ME: masticatory efficiency; MO: mouth opening; MRI: magnetic resonance imaging; *n*: number; OM: oral motor; PDP: physiotherapeutic and drug protocol; PE: protrusion excursion; PI: pain intensity; PPT: pressure pain threshold; SF-MPQ: short form McGill Pain Questionnaire; TMD:_temporomandibular disorder; TMJ:_temporomandibular joint; VAS: visual analogue scale.

**Table 2:** Parameters of LLLT and outcomes in the included trials.

Study	Laser type	Treatment time/number of total sessions/number of sessions week^−1^	Application sites	Power	Dosage (J/cm^2^)	Outcome
Conti [17]	GaAIAs 830 nm	40 s/3/1	TMJ and/or muscles	79 mW	4	LLLT = placebo
Kulekcioglu [49]	GaAs 904 nm	180 s/15/–	TMJ and/or muscles	17 mW	3	LLLT > placebo (MO, LM)
						LLLT = placebo (PI, TMJ sounds)
Venancio [19]	GaAlAs 780 nm	10 s/6/2	TMJ	30 mW	6.3	LLLT = placebo
Mazzetto [41]	GaAIAs 780 nm	10 s/8/2	TMJ (external auditory meatus)	70 mW	89.7	LLLT > placebo
Cunha [43]	GaAlAs 830 nm	20 s/4/1	TMJ and/or muscles	500 mW	100	LLLT = placebo
Carrasco [1]	GaAlAs 780 nm	60 s/8/2	TMJ	70 mW	105	LLLT > placebo (PI on palpation) LLLT = placebo (ME: masticatory efficiency)
Emshoff [18]	HeNe 632.8 nm	120 s/20/2-3	TMJ	30 mW	1.5	LLLT = placebo
Lassemi [50]	GaAs 980 nm	60 s/2/2	TMJ and muscles	NA	NA	LLLT > placebo
Carrasco [43]	GaAlAs 780 nm	60 s/8/2	Muscles	50/60/70 mW	25/60/105	LLLT = placebo
Shirani [53]	InGaAlP 660 nm and GaAs 890 nm	360 s/6/2	Muscles	17.3 mW and 1.76 mW	6.2 and 1.0	LLLT > placebo
		600 s/6/2				
Marini [20]	GaAIAs 910 nm	20 min/10/5	TMJ	400 mW	NA	LLLT > placebo
Sattayut [35]	GaAIAs 820 nm	–/3/–	TMJ and/or muscles	60 mW or 300 mW	21.4 or 107	LLLT > placebo (high energy)
						LLLT = placebo (low energy)
Silva [36]	GaAIAs 780 nm	30 s or 60 s/10/2	TMJ and/or muscles	70 mW	52.5 or 105.0	LLLT > placebo
Ferreira [16]	GaAIAs 780 nm	90 s/12/1	TMJ and muscles	50 mW	112.5	LLLT > placebo
Ahrari [44]	GaAIAs 810 nm	120 s/12/3	Muscles	50 mW	3.4	LLLT > placebo
Demirkol [54]	Nd:YAG 1064 nm	20 s/10/5	Muscle	250 mW	8	LLLT > placebo
Röhlig [51]	GaAs 820 nm	10 s/10/3-4	Muscle	300 mW	8	LLLT > placebo
Wang [21]	GaAIAs 650 nm/830 nm	15 min/6/6	TMJ	300 mW	NA	LLLT > placebo
Carli [28]	GaAlAs 830 nm	28 s/4/2	TMJ and muscles	100 mW	100	LLLT = placebo
Fornaini [31]	GaAlAs 808 nm	15 min/14/7	TMJ	250 mW	NA	LLLT > placebo
Sancakli [30]	GaAs 820 nm	10 s/12/3	Muscle	300 mW	3	LLLT > placebo
Frare [52]	GaAs 904 nm	16 s/8/2	TMJ and external auditory meatus	15 mW	6	LLLT > placebo
Venezian [45]	GaAIAs 780 nm	20 or 40 s/8/2	Muscles	50/60 mW	25 or 60	LLLT = placebo
Mazzetto [46]	GaAlAs 830 nm	10 s/8/2	TMJ	40 mW	5	LLLT > placebo
Uemoto [55]	Laser type NA, 795 nm	–/4/–	Muscle	80 mW	4 or 8	LLLT > placebo (only 4 J/cm^2^)
Madani [56]	Laser type NA, 810 nm	120 s/12/3	TMJ and muscles	50 mW	3.4	LLLT = placebo
Maia [29]	GaAlAs 808 nm	19 s/8/2	Muscle	100 mW	70	LLLT > placebo
Cavalcanti [47]	GaAlAs780 nm	20 s/12/3	TMJ and muscles	70 mW	35.0	LLLT > placebo
Magri [48]	GaAlAs 780 nm	10 s/8/2	TMJ and muscles	TMJ, 20 mW; muscle, 30 mW	5 or 7.5	LLLT = placebo
Demirkol [4]	Nd:YAG laser (1064 nm), diode laser (810 nm)	20 s or 9 s/10/5	External auditory meatus	250 mW	8	LLLT > placebo
Machado [40]	GaAlAs 780 nm	45 min/12/1–0.5	TMJ and muscles	60 mW	60 ± 1.0	LLLT = placebo

GaAlAs: gallium-aluminium-arsenide laser; Ga-Ar: gallium argon; GaAs: gallium-arsenide laser; HeNe: helium-neon laser; InGaAlP: indium-gallium-aluminum-phosphide laser; LLLT: low-level laser therapy; NA: not available; Nd:YAG: neodymium-doped yttrium aluminum garnet; TMJ: temporomandibular joint.

## Supplementary Materials

Supplementary Materials Appendix A: quality assessment of the included studies according to modified jadad score. Appendix B: forest plots of primary outcomes (pain). Appendix C: primary outcomes/pain-sensitivity analysis. Appendix D: forest plots (functional outcomes). Appendix E: funnel plot. Appendix F: GRADE evidence profile for pain and functional outcomes.
