# Supplementary material for: Low-Level Laser Therapy for Temporomandibular Disorders: A Systematic Review with Meta-Analysis
Source: Pain Res Manag. 2018 May 10;2018:4230583. doi: 10.1155/2018/4230583 (PMC5971344; doi:10.1155/2018/4230583)
Supplement: Supplementary Materials — Appendix A: quality assessment of the included studies according to modified jadad score. Appendix B: forest plots of primary outcomes (pain). Appendix C: primary outcomes/pain-sensitivity analysis. Appendix D: forest plots (functional outcomes). Appendix E: funnel plot. Appendix F: GRADE evidence profile for pain and functional outcomes. [file 4230583.f1.docx]

Content

Appendix A: Quality Assessment of the Included Studies According to Modified Jadad score

Appendix B: Forest plots of primary outcomes (pain)

Appendix C: Primary outcomes / Pain- sensitivity analysis

Appendix D: Forest Plots (functional outcomes)

Appendix E: Funnel Plot

Appendix F: GRADE evidence profile for pain and functional outcomes

Appendix A

Quality Assessment of the Included Studies According to Modified Jadad score

| Study | Was the study described as randomized? | Was the method of randomization appropriate? | Was the study described as blinded? | Was the method of blinding appropriate? | Was there a description of withdrawals and dropouts? | Was there a clear description of the inclusion/exclusion criteria? | Was the method used to assess adverse effects described？ | Was the method of statistical analysis described? | Modified Jadad Score |
| --- | --- | --- | --- | --- | --- | --- | --- | --- | --- |
| Conti 1997 | 1 | 0 | 1 | +1 | 1 | 0 | 0 | 1 | 5 |
| De Carli 2013 | 1 | +1 | 1 | +1 | 1 | 1 | 1 | 1 | 8 |
| Emshoff 2008 | 1 | +1 | 1 | +1 | 1 | 1 | 0 | 1 | 7 |
| Ahrari 2014 | 1 | 0 | 1 | +1 | 1 | 1 | 0 | 1 | 6 |
| Carrasco 2008 | 1 | 0 | 1 | +1 | 0 | 1 | 0 | 1 | 5 |
| Ferreira 2013-R | 1 | +1 | 1 | +1 | 0 | 1 | 1 | 1 | 7 |
| Fornaini 2015 | 1 | 0 | 1 | +1 | 0 | +1 | 0 | 1 | 5 |
| Frare 2008 | 1 | -1 | 1 | 0 | 1 | 0 | 1 | 1 | 4 |
| Kulekcioglu 2003 | 1 | 0 | 1 | +1 | 0 | 1 | 0 | 1 | 5 |
| Lassemi 2008 | 1 | 0 | 1 | -1 | 1 | 0 | 0 | 1 | 3 |
| Marini 2010-R | 1 | 0 | 1 | +1 | 1 | 1 | 0 | 1 | 6 |
| Mazzetto 2007-R | 1 | 0 | 1 | +1 | 1 | 1 | 0 | 1 | 6 |
| Mazzetto 2010 | 1 | 0 | 0 | 0 | 1 | 1 | 0 | 1 | 4 |
| Carrasco 2009-R | 1 | 0 | 1 | +1 | 0 | 1 | 0 | 1 | 5 |
| Da Cunha 2008 | 1 | 0 | 1 | +1 | 0 | 1 | 0 | 1 | 5 |
| Demirkol 2014 | 1 | 0 | 0 | 0 | 0 | 1 | 1 | 1 | 4 |
| Venancio 2005 | 1 | 0 | 1 | +1 | 0 | 1 | 0 | 1 | 5 |
| Shirani 2009 | 1 | 0 | 1 | +1 | 0 | 1 | 1 | 1 | 6 |
| Venezian 2010 | 1 | +1 | 1 | +1 | 0 | 1 | 0 | 1 | 6 |
| Sattayut 2012 | 1 | 0 | 1 | +1 | 1 | 1 | 0 | 1 | 6 |
| Silva 2012 | 1 | 0 | 1 | +1 | 1 | 1 | 0 | 1 | 6 |
| Moraes Maia 2014 | 1 | 0 | 1 | 0 | 1 | 1 | 0 | 1 | 5 |
| Röhlig 2011 | 1 | +1 | 1 | +1 | 1 | 1 | 1 | 1 | 8 |
| Wang 2011 | 1 | 0 | 1 | +1 | 0 | 1 | 0 | 1 | 5 |
| Uemoto 2013 | 1 | 0 | 0 | 0 | 1 | 1 | 0 | 1 | 4 |
| Machado 2015 | 1 | +1 | 1 | -1 | 1 | 1 | 0 | 1 | 5 |
| Sancakli 2015 | 1 | +1 | 1 | +1 | 0 | 1 | 1 | 1 | 7 |
| Madani 2014 | 1 | 0 | 1 | +1 | 1 | 1 | 0 | 1 | 6 |
| Cavalcanti 2016 | 1 | 0 | 0 | 0 | 0 | 1 | 1 | 1 | 4 |
| Magri 2017 | 1 | +1 | 1 | +1 | 1 | 1 | 0 | 1 | 7 |
| Demirkol 2017 | 1 | 0 | 1 | -1 | 0 | 1 | 1 | 1 | 4 |

Appendix B – Forest plots of outcome measures (pain)

## 1. Effects on VAS scores

1.1. Forest plot analysis of the VAS score at the final follow-up time point

A

Figure 1 Subgroup analysis: low dose ≤50 J/cm^2^ versus high dose >50 J/cm^2^

B

Figure 2 Subgroup analysis: short-term follow-up (≤2 weeks) versus long-term follow-up (>2 weeks)

2. Effects on change in VAS scores

2.1 Forest plot analysis of the pooled weighted mean differences for VAS score

A

Figure 3 Subgroup analysis: low dose ≤50 J/cm2 versus high dose >50 J/cm2

B

Figure 4 Subgroup analysis: short-term follow-up (≤2 weeks) versus long-term follow-up (>2 weeks)

Appendix C

Primary outcome pain effect sizes

a. Subgroup analysis at VAS and Change of VAS score

b. Sensitivity analysis (without Lassemi 2008 and Marini 2010)

| Subgroups | VAS score | | | | Change of VAS score | | | |
| --- | --- | --- | --- | --- | --- | --- | --- | --- |
|  | trials (n) | WMD [mm] (95%CI) | χ^2^  (p)  [I^2^] | WMD (95%CI) Sensitivity analysis (without Lassemi 2008 and Marini 2010) | trials (n) | WMD [mm] (95%CI) | χ^2^  (p)  [I^2^] | WMD (95%CI) Sensitivity analysis (without Lassemi 2008 and Marini 2010) |
| total | 17(643) | -14.05(-25.67, -2.43) | 534.63  (<0.00001)  [96%] | -11.88(-18.27, -5.49) | 19(679) | 15.43(3.61, 27.26) | 1240.82  (<0.00001)  [98%] | 13.19(6.49, 19.89) |
| low dose (≤50 J/cm^2^) | 8(242) | -9.22(-18.78, 0.34) | 53.54  (<0.00001)  [85%] | -9.22(-18.78, 0.34) | 10(278) | 15.09(5.37, 24.80) | 138.30  (<0.00001)  [93%] | 15.09(5.37, 24.80) |
| high dose  (>50 J/cm^2^) | 7(218) | -10.42(-19.67, -1.17) | 14.19  (=0.05)  [51%] | -10.42(-19.67, -1.17) | 7(218) | 5.52(-5.52, 16.56) | 35.10  (<0.0001)  [80%] | 5.52(-5.52, 16.56) |
| dose unknow | 4(183) | -33.75(-57.18, -10.33) | 120.62  (<0.00001)  [98%] | -27.04(-33.72, -20.35) | 4(183) | 36.31(10.63, 61.98) | 306.15  (<0.00001)  [99%] | 28.81(24.58, 33.03) |
| short-term (≤2 weeks) | 8(304) | -14.66(-21.04, -8.29) | 31.16  (=0.0003)  [71%] | -13.83(-21.03, -6.62) | 10(340) | 17.66(9.94, 25.38) | 113.11  (<0.00001)  [90%] | 17.23(8.74,25.72) |
| long-term (>2 weeks) | 9(339) | -14.84(-35.35,5.68) | 338.59  (<0.00001)  [97%] | -10.16(-22.07, 1.76) | 9(339) | 13.85(-7.73, 35.43) | 694.10  (<0.00001)  [99%] | 8.69(-3.42, 20.81) |

Appendix D. Effects on functional scores

1. Mean differences for the maximum active mouth opening (MAVO) at the final follow-up time point

A

Figure 5 Subgroup analysis: low dose ≤50 J/cm2 versus high dose >50 J/cm2

B

Figure 6 Subgroup analysis: short-term follow-up (≤2 weeks) versus long-term follow-up (>2 weeks)

2. Mean differences for the maximum passive mouth opening (MPVO) at the final follow-up time point

A

Figure 7 Subgroup analysis: low dose ≤50 J/cm2 versus high dose >50 J/cm2

B

Figure 8 Subgroup analysis: short-term follow-up (≤2 weeks) versus long-term follow-up (>2 weeks)

3. Mean differences for lateral excursion (LE) at the final follow-up time point

A

Figure 9 Subgroup analysis: low dose ≤50 J/cm2 versus high dose >50 J/cm2

B

Figure 10 Subgroup analysis: short-term follow-up (≤2 weeks) versus long-term follow-up (>2 weeks)

4. Mean differences for protrusion excursion (PE) at the final follow-up time point

A

Figure 11 Subgroup analysis: low dose ≤50 J/cm2 versus high dose >50 J/cm2

B

Figure 12 Subgroup analysis: short-term follow-up (≤2 weeks) versus long-term follow-up (>2 weeks)

Appendix E

Funnel Plot


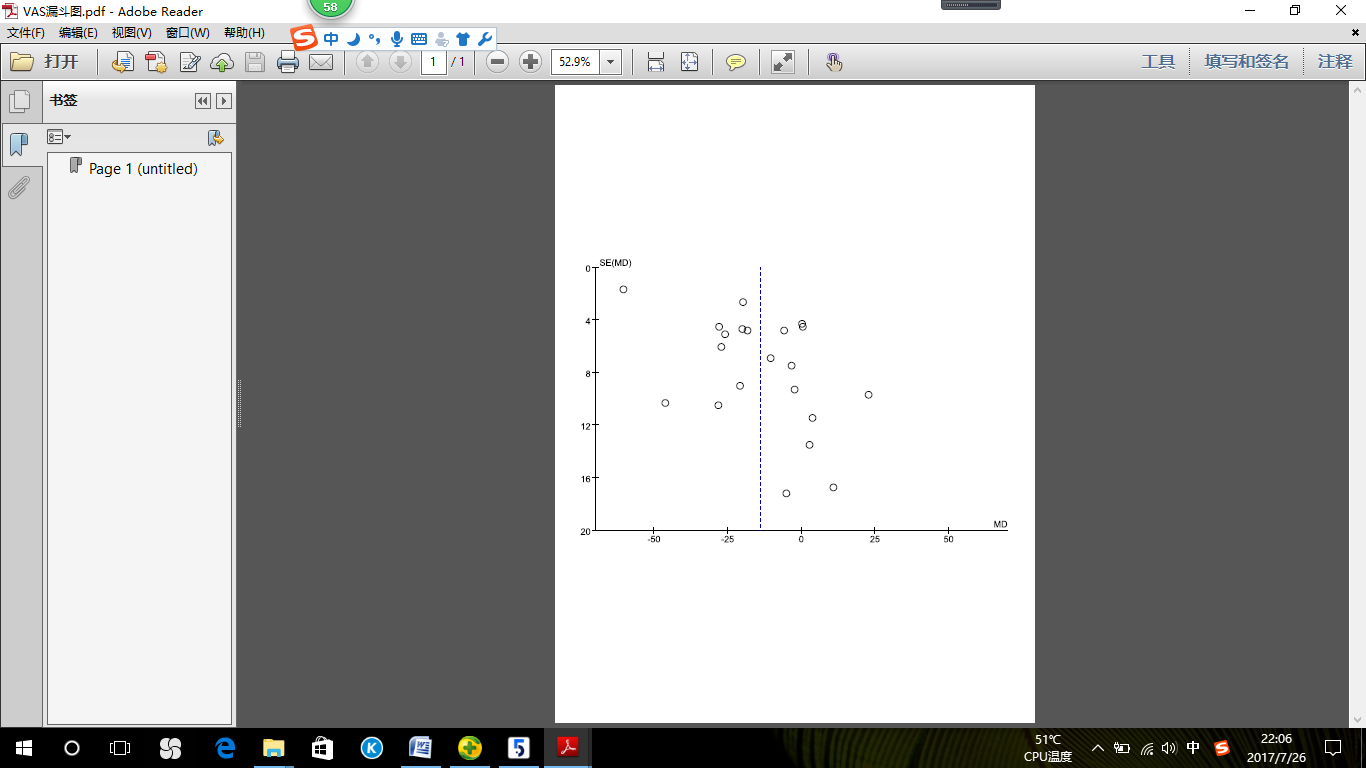


Figure 13 Funnel plot of comparison Laser versus placebo, outcome: Pain (VAS score). Studies with positive results are towards the left.


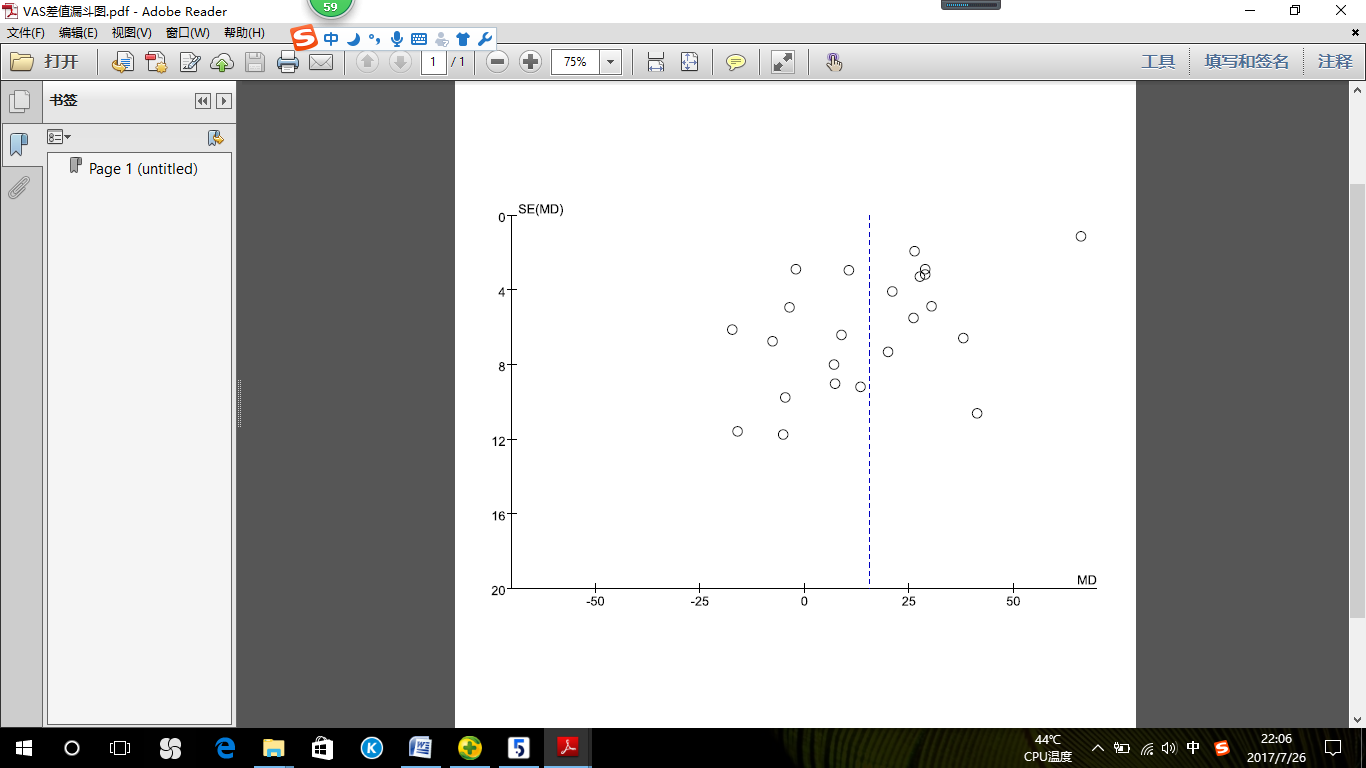


Figure 14 Funnel plot of comparison Laser versus placebo, outcome: Pain (change of VAS score). Studies with positive results are towards the right.

Appendix F

GRADE evidence profile for primary outcomes

| Outcomes | number of studies (pooled sample) | test for heterogeneity | | analytic model | test for overall effect | | MD | 95%CI of MD | GRADE |
| --- | --- | --- | --- | --- | --- | --- | --- | --- | --- |
|  |  | I*^2^* | p |  | Z | p |  |  |  |
| VAS score | 17(643) | 96% | <0.00001 | Random | 2.37 | 0.02 | -14.05 | -25.67, -2.43 |  |
| low dose group | 8(242) | 85% | <0.00001 | Random | 1.89 | 0.06 | -9.22 | -18.78, 0.34 | Very low⊕OOO |
| high dose group | 7(218) | 51% | 0.05 | Random | 2.21 | 0.03 | -10.42 | -19.67, -1.17 | Low⊕ ⊕ OO |
| dose unknow group | 4(183) | 98% | <0.00001 | Random | 2.82 | 0.005 | -33.75 | -57.18, -10.33 | Very low⊕OOO |
| short-term group | 8(304) | 71% | =0.0003) | Random | 4.51 | <0.00001 | -14.66 | -21.04, -8.29 | Low⊕⊕OO |
| long-term group | 9(339) | 97% | <0.00001 | Random | 1.42 | 0.16 | -14.84 | -35.35,5.68 | Very low⊕OOO |
| Change of VAS score | 19(679) | 98% | <0.00001 | Random | 2.56 | 0.01 | 15.43 | 3.61, 27.26 |  |
| low dose group | 10(278) | 93% | <0.00001 | Random | 3.04 | 0.002 | 15.09 | 5.37, 24.80 | Very low⊕OOO |
| high dose group | 7(218) | 80% | <0.0001 | Random | 0.98 | 0.33 | 5.52 | -5.52, 16.56 | Low⊕⊕OO |
| dose unknow group | 4(183) | 99% | <0.00001 | Random | 2.77 | 0.006 | 36.31 | 10.63, 61.98 | Low⊕⊕OO |
| short-term group | 10(340) | 90% | <0.00001 | Random | 4.48 | <0.00001 | 17.66 | 9.94, 25.38 | Low⊕⊕OO |
| long-term group | 9(339) | 99% | <0.00001 | Random | 1.26 | 0.21 | 13.85 | -7.73, 35.43 | Very low⊕OOO |
| MAVO | 8(301) | 95% | <0.00001 | Random | 3.52 | 0.0004 | 6.37 | 2.82, 9.93 |  |
| low dose group | 5(145) | 95% | <0.00001 | Random | 1.73 | 0.08 | 6.41 | -0.84, 13.66 | Very low⊕OOO |
| high dose group | 1(45) | 0% | 0.47 | Random | 4.66 | <0.00001 | 4.18 | 2.42, 5.94 | Low⊕⊕OO |
| dose unknow group | 2(111) | 91% | 0.0009 | Random | 3.64 | 0.0003 | 8.09 | 3.73, 12.45 | Very low⊕OOO |
| short-term group | 2(60) | 98% | <0.00001 | Random | 1.13 | 0.26 | 8.32 | -6.16, 22.80 | Very low⊕OOO |
| long-term group | 6(241) | 84% | <0.00001 | Random | 4.36 | <0.0001 | 5.79 | 3.18, 8.39 | Low⊕⊕OO |
| MPVO | 3(144) | 92% | <0.00001 | Random | 2.74 | 0.006 | 6.96 | 1.99, 11.93 |  |
| low dose group | 2(75) | 0% | 0.57 | Random | 4.59 | <0.00001 | 4.96 | 2.84, 7.07 | Low⊕⊕OO |
| high dose group | -- | -- | -- | Random | -- | -- | -- | -- | -- |
| dose unknow group | 1(69) | -- | -- | Random | 18.08 | <0.00001 | 10.97 | 9.78,12.16 | Low⊕⊕OO |
| short-term group | 1(40) | -- | -- | Random | 4.50 | <0.00001 | 5.18 | 2.93, 7.43 | Low⊕⊕OO |
| long-term group | 2(104) | 83% | 0.02 | Random | 2.05 | 0.04 | 7.75 | 0.33, 15.17 | Very low⊕OOO |
| LE | 6(477) | 90% | <0.00001 | Random | 7.78 | <0.00001 | 3.52 | 2.63, 4.40 |  |
| low dose group | 3(210) | 95% | <0.00001 | Random | 2.69 | 0.007 | 2.76 | 0.75, 4.77 | Very low⊕OOO |
| high dose group | 1(45) | 65% | 0.09 | Random | 4.81 | <0.00001 | 4.38 | 2.59, 6.16 | Moderate⊕⊕⊕O |
| dose unknow group | 2(222) | 80% | 0.002 | Random | 9.78 | <0.00001 | 4.05 | 3.24, 4.87 | Moderate⊕⊕⊕O |
| short-term group | 1(80) | 0% | 0.66 | Random | 22.85 | <0.00001 | 5.08 | 4.64, 5.52 | Moderate⊕⊕⊕O |
| long-term group | 5(397) | 90% | <0.00001 | Random | 5.61 | <0.00001 | 3.12 | 2.03, 4.22 | Very low⊕OOO |
| PE | 4(157) | 95% | <0.00001 | Random | 2.06 | 0.04 | 1.77 | 0.09, 3.45 |  |
| low dose group | 2(70) | 97% | <0.00001 | Random | 1.22 | 0.22 | 2.27 | -1.38, 5.92 | Very low⊕OOO |
| high dose group | 1(45) | 0% | 1.00 | Random | 5.17 | <0.00001 | 2.13 | 1.32, 2.94 | Moderate⊕⊕⊕O |
| dose unknow group | 1(42) | -- | -- | Random | 0.27 | 0.78 | 0.09 | -0.55, 0.73 | Moderate⊕⊕⊕O |
| short-term group | 1(40) | -- | -- | Random | 10.92 | <0.00001 | 4.12 | 3.38, 4.86 | Moderate⊕⊕⊕O |
| long-term group | 3(117) | 81% | 0.001 | Random | 2.03 | 0.04 | 1.11 | 0.04, 2.18 | Low⊕⊕OO |
